# Precise Tuning of Facile One-Pot Gelatin Methacryloyl (GelMA) Synthesis

**DOI:** 10.1038/srep31036

**Published:** 2016-08-09

**Authors:** Hitomi Shirahama, Bae Hoon Lee, Lay Poh Tan, Nam-Joon Cho

**Affiliations:** 1 School of Materials Science and Engineering, Nanyang Technological University, 50 Nanyang Avenue, 639798 Singapore; 2 School of Chemical and Biomedical Engineering, Nanyang Technological University, 62 Nanyang Drive, 637459 Singapore

## Abstract

Gelatin-methacryloyl (GelMA) is one of the most commonly used photopolymerizable biomaterials in bio-applications. However, GelMA synthesis remains suboptimal, as its reaction parameters have not been fully investigated. The goal of this study is to establish an optimal route for effective and controllable GelMA synthesis by systematically examining reaction parameters including carbonate-bicarbonate (CB) buffer molarity, initial pH adjustment, MAA concentration, gelatin concentration, reaction temperature, and reaction time. We employed several analytical techniques in order to determine the degree of substitution (DS) and conducted detailed structural analysis of the synthesized polymer. The results enabled us to optimize GelMA synthesis, showing the optimal conditions to balance the deprotonation of amino groups with minimizing MAA hydrolysis, which led to nearly complete substitution. The optimized conditions (low feed ratio of MAA to gelatin (0.1 mL/g), 0.25 M CB buffer at pH 9, and a gelatin concentration of 10–20%) enable a simplified reaction scheme that produces GelMA with high substitution with just one-step addition of MAA in one pot. Looking forward, these optimal conditions not only enable facile one-pot GelMA synthesis but can also guide researchers to explore the efficient, high methacrylation of other biomacromolecules.

Gelatin is an attractive biomaterial that is obtained from the partial hydrolysis of collagen, the most abundant protein in the human body^1^. It is biocompatible, biodegradable, and suitable for a wide range of cell types. Gelatin can provide adequate cell attachment via RGD (Arg-Gly-Asp) motifs^2^, and it plays an important role in cell proliferation^3^, function^4^, and differentiation^5^. Furthermore, gelatin is less immunogenic than collagen^6^,^7^ due to the reduced presence of aromatic groups^8^. In addition, gelatin is relatively easy to obtain and low in cost compared with other natural materials.

Raw gelatin can only form a physical hydrogel at specific concentrations and temperatures, albeit with low mechanical strength. To improve hydrogel stiffness, a number of crosslinking strategies have been adopted, including the use of crosslinking chemicals (e.g., glutaraldehyde^9^ and genipin^10^) and chemical modification to support photo-crosslinking (e.g., methacrylic anhydride [MAA]). Compared with the use of crosslinking chemicals, photo-crosslinking methods provide fast, uniform *in situ* curing. Among the chemicals used for gelatin modification, MAA is the most widely used. The product, gelatin methacryloyl (GelMA), has been used in various bio-applications (e.g., micropatterning^11^,^12^, fluidic systems^13^,^14^, 3D scaffolds^15^,^16^, bioprinting^17^,^18^,^19^,^20^) with different cells (e.g., fibroblasts^11^,^21^,^22^, stem cells^23^,^24^, cartilage^17^,^25^, hepatocytes^19^,^26^) and composite materials (e.g., carbon nanotubes^27^, graphene oxide^28^,^29^, natural polymers^30^,^31^, synthetic polymers^32^,^33^).

The method of synthesizing GelMA was originally developed by Van Den Bulcke *et al*. in ref. 34. Briefly, MAA monomers were reacted with lysine and hydroxyl lysine groups of gelatin (Fig. 1A) by dissolving gelatin in phosphate-buffered saline (PBS) solution at 50 °C (Fig. 1B). This original report opened up a new arena for GelMA in biomaterial research and tissue engineering applications. However, GelMA synthesis routes remain suboptimal, leaving considerable room for improvement, especially in terms of controllability and efficacy^35^. For example, in theory, one MAA molecule could react with one lysine group. Nevertheless, studies following the original method have reported using MAA concentrations 18–47 times higher than that of gelatin in order to obtain high percentages of gelatin methacryloyl product, expressed as degree of substitution (DS) (>85%)^36^,^37^,^38^,^39^,^40^,^41^ (cf., Supplementary Figure S1).

Various attempts have been made in order to enhance the synthesis scheme to improve reaction efficacy. For example, Martineau *et al*. used a water-miscible organic chemical (dimethyl sulfoxide, DMSO) as the solvent choice rather than PBS^42^. This method effectively hindered MAA’s contact with water, which can result in hydrolysis, and improved the DS as compared to the use of PBS. However, it requires an organic base and an additional step of precipitation with ethanol, which leads to a low yield^42^,^43^.

Another means of synthesis is to employ pH adjustment during synthesis in order to keep the free amino groups neutral to react with MAA. The essence of this method is to maintain the pH of the reaction solution above the isoelectric point (IEP) of gelatin, keeping the free amino groups of lysine neutral to allow them to react with MAA. The IEP differs for different types of gelatin: 8–9 for type A and 5–6 for type B^44^. The use of PBS (pH 7.4) as a buffer is not sufficient for pH maintenance because a byproduct of the reaction, methacrylic acid, alters the pH, rendering it acidic. Although pH adjustment leads to improved efficacy, it still requires a 10–32 molar excess of MAA^45^,^46^,^47^. In addition, manual pH adjustment is laborious, and the resulting DS is highly dependent on the operating technique.

We recently reported a more effective method that involves sequentially adjusting the pH followed by MAA addition during the reaction^48^. In addition, it has been demonstrated that the use of carbonate-bicarbonate (CB) buffer can lead to a higher DS in comparison to the use of PBS. In conjunction with sequential manipulation, the use of CB buffer achieved nearly complete substitution (97%) and dramatically reduced the excess MAA molar ratio down to 2.2-fold from the conventional 10- to 32-fold excess^45^,^46^,^47^. Based on these findings, we hypothesized that a higher CB molarity synthesis scheme can be further simplified by using a high pH that is sufficiently above the IEP of gelatin in order to achieve a complete reaction of MAA with free amino groups of gelatin. Such a scheme might overcome the need for the laborious sequential process of pH adjustment and MAA addition. Importantly, to the best of our knowledge, there is still no comprehensive understanding of the influence of how reaction parameters such as gelatin concentration, reaction temperature and molar ratio influence the methacrylation of biomacromolecules. Towards this goal, we report here a systematic investigation leading to the identification of optimal reaction parameters for controlling the GelMA synthesis scheme. Precise tuning of the reaction parameters can yield a more effective scheme in terms of cost, energy, time, and labor, as compared to conventional GelMA synthesis routes.

## Results and Discussion

**Figure 1:**
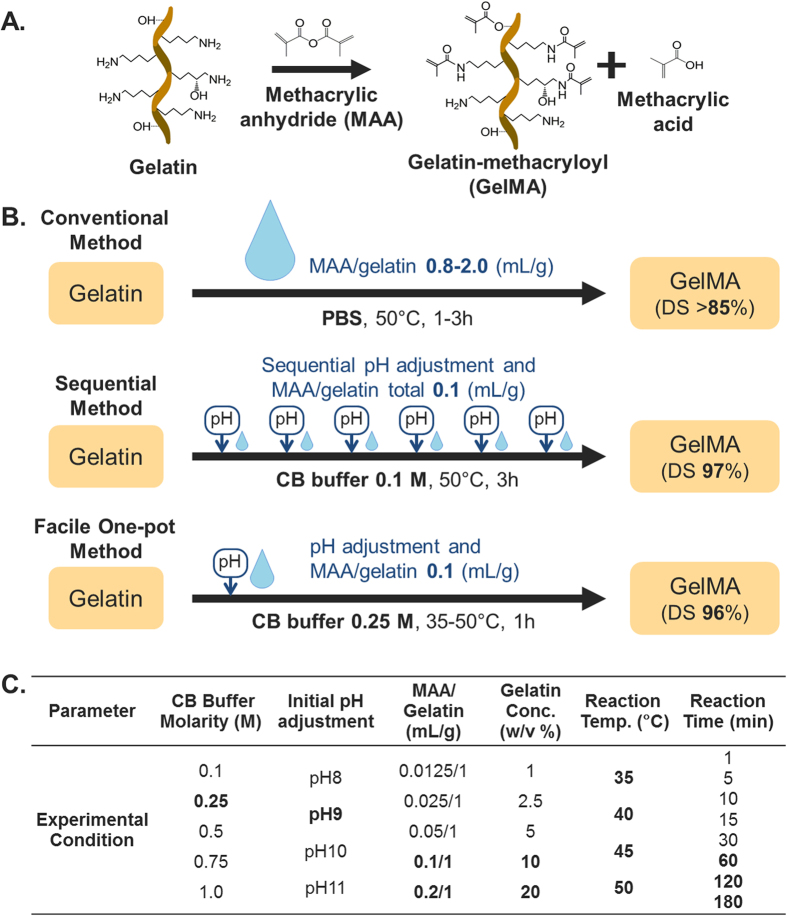
(**A**) Schematic illustration of gelatin-methacryloyl (GelMA) synthesis. (**B**) Schematic illustration of different synthesis processes of GelMA and their respective degrees of substitution. The conventional method requires a large amount of methacrylic anhydride (MAA). Although sequential processing reduces MAA consumption, multiple pH adjustments are needed, being followed by MAA addition. Our proposed method, *Facile One-pot Synthesis*, minimizes both MAA consumption and manual work. (**C**) Experimental parameters and optimized conditions for synthesis. ^a^PBS: Phosphate-buffered saline, ^b^DS: Degree of substitution, ^c^CB: Carbonate-bicarbonate, ^d^Optimum in bold.

**Figure 2:**
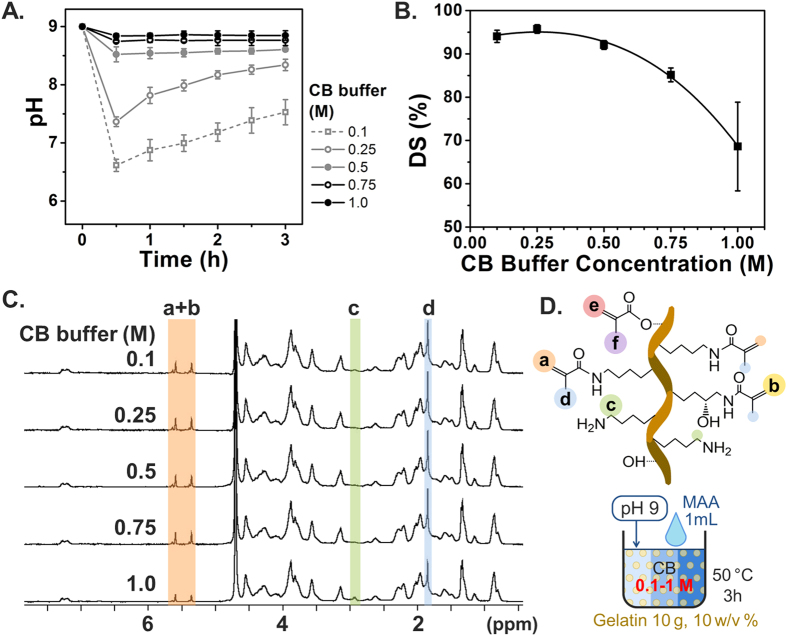
Effect of different CB molarities on DS of GelMA synthesis. Error bars indicate the relative standard deviations of three or more different samples (n ≥ 3). (**A**) pH transition kinetics during the reaction. (**B**) DS versus CB molarity. DS was obtained from TNBS assay. A higher CB buffer concentration kept pH more steady but led to a lower DS. The highest DS was obtained at 0.25 M CB. (**C**) ^1^H NMR verification. Peaks correspond to acrylic protons (2H) of methacrylamide grafts of lysine groups (a) and those of hydroxyl lysine groups (b), methylene protons (2H) of unreacted lysine groups (c), methyl protons (3H) of methacrylamide grafts (d), acrylic protons (2H) of methacrylated grafts of hydroxyl groups (e), and methyl protons (3H) of methacrylated grafts of hydroxyl groups (f). (**D**) Schematic illustration of GelMA corresponding to ^1^H NMR peaks.

To precisely control GelMA synthesis with maximum DS, we first altered the molar concentration of CB buffer, which is comprised of sodium carbonate and sodium bicarbonate as shown in Fig. 2. The control range of the CB molar concentration was set from 0.1 to 1 M, with the aim of maintaining the pH at a level higher than that of the IEP of type A gelatin during the reaction. Further, we initially adjusted the pH to 9 prior to the reaction with MAA. The gelatin solution was prepared at a concentration of 10 w/v%, and 0.1 mL of MAA per gram of gelatin was used, the same conditions as those used in our previous study (Fig. 1B). This MAA amount was calculated to be a 2.2-fold molar excess over the free amino group of gelatin, with reference to the literature (0.286 mmol of amino groups per gram of gelatin)^49^. The pH changes in each reaction solution were monitored every 30 min for 3 h during the reaction at 50 °C, as shown in Fig. 2A. During synthesis, reaction solutions with a higher CB buffer concentration had a greater buffering capacity and hence were able to maintain more closely the original pH up to completion of the reaction scheme. However, in both the 0.1 M and 0.25 M CB buffer cases, we observed a sharp drop in pH to 6.6 during the initial reaction, signifying that the reaction took place within a short period of time. Furthermore, after 30 min of reaction, we observed a slight increase in pH, which suggests that the reaction might be completed, and that the pH of the solution was being restored by the CB buffer capacity. To measure DS as a function of CB buffer concentration, a 2,4,6-trinitrobenzene-sulfonic acid (TNBS) assay was conducted after completion of the reaction. The results showed that the DS decreaseed significantly with an increase in the CB buffer concentration, particularly above 0.5 M CB buffer solution. This supports that the hydrolysis of MAA is further accelerated in CB buffer solutions with a higher molarity (Fig. 2B). This trend was also observed in experimental results obtained by proton nuclear magnetic resonance (^1^H NMR) analysis, as shown in Fig. 2C. The peaks of methylene lysine protons (2H) around 2.8 ppm (peak c) did not appear in the spectra with a high DS, indicating the complete conjugation of lysine with MAA. Further, the acrylic protons (2H) of the methacrylamide grafts around 5.5 ppm (peak a + b) and those of the methyl protons (3H) of methacrylamide around 1.9 ppm (peak d) were higher in the higher DS samples. Nearly complete substitution (DS = 95.75 ± 0.98%) was achieved at a CB buffer concentration of 0.25 M, which is the highest DS among the experimental groups. The polynomial fitting curves of the data points suggest a local maximum value at 0.257 M, which implies that 0.25 M CB buffer is close to the optimal buffer concentration. This concentration was therefore selected for further investigation.

**Figure 3:**
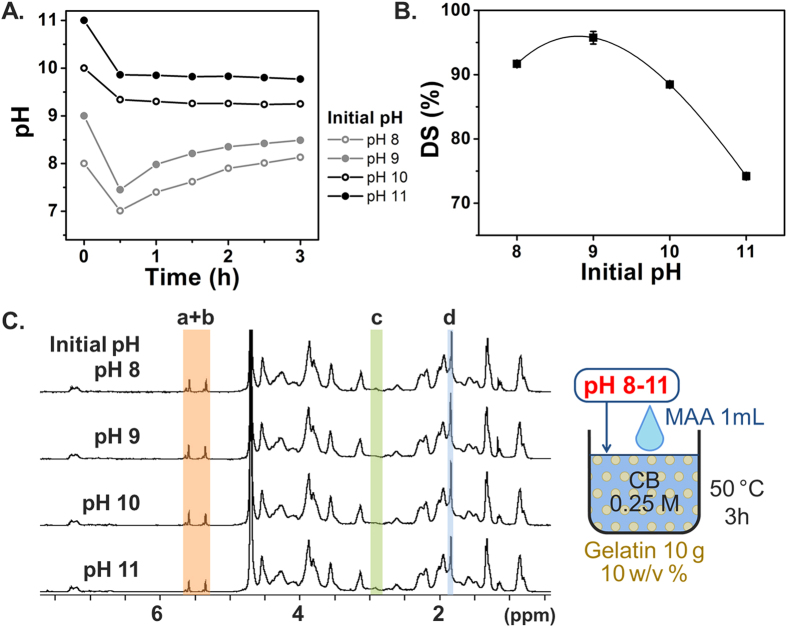
Effect of different initial pH adjustments on DS of GelMA synthesis. Error bars indicate the relative standard deviations of three independent measurements (n = 3). (**A**) pH transition kinetics during the reaction. (**B**) DS versus initial pH. DS was obtained from TNBS assay. The highest DS was produced at pH 9 in 0.25 M CB (**C**) ^1^H NMR verification. Peaks correspond to acrylic protons (2H) of methacrylamide grafts of lysine groups (a) and those of hydroxyl lysine groups (b), methylene protons (2H) of unreacted lysine groups (c), and methyl protons (3H) of methacrylamide grafts (d).

We then investigated the optimal pH for the initial pH adjustment step in order to further optimize the synthesis conditions. In the preceding set of experiments that identified the dependence on CB buffer concentration, the pH of the gelatin solution was adjusted to 9 prior to MAA addition. In this next set, different initial pH adjustments were conducted, including pH 8, 9, 10, and 11, in order to determine the optimal initial pH adjustment condition based on determining which one yielded the highest DS. The pH transitions in Fig. 3A show that 0.25 M CB buffer stabilized the pH around pH 8–10 as the reaction progressed. The corresponding DS results in Fig. 3B show that the initial pH adjustment step to pH 9 led to a higher DS than the reactions conducted with other pH adjustment steps above or below this optimal value (pH 8, 10 and 11). This finding indicates that reactions at a lower pH (pH 8) may be hindered by greater protonation of the free amino groups, whereas those at a higher pH (pH 10 and 11) may be hampered by excess MAA hydrolysis catalyzed by a strong base (hydroxide ion)^48^. The polynomial fitting curve reached a local maximum value at pH 8.8, which implies that initial adjustment to pH 9 is optimal among the test cases. As shown in Fig. 3C, the TNBS results agree well with the ^1^H NMR results. Taken together, these findings support that initial pH adjustment to 9 with 0.25 M CB buffer creates the optimal conditions for balancing the deprotonation of amino groups with MAA hydrolysis.

**Figure 4:**
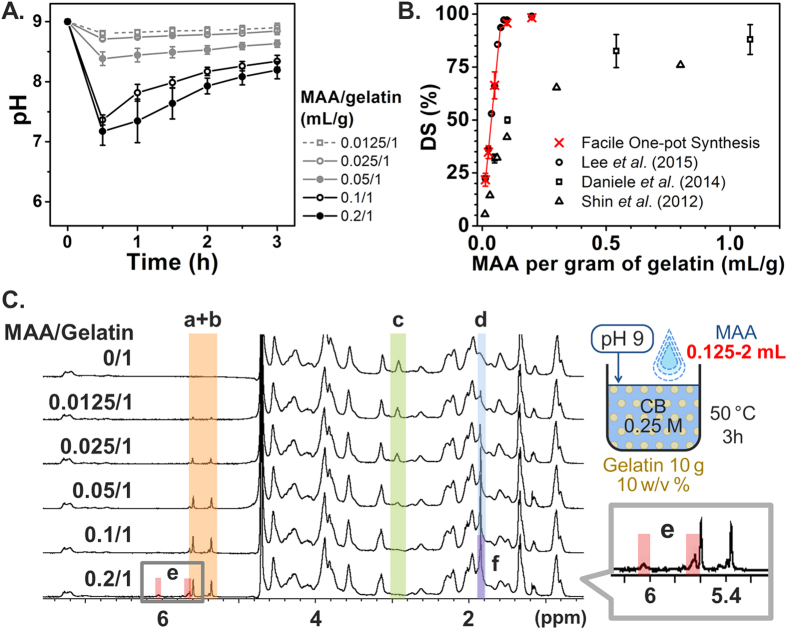
Effect of MAA/gelatin ratios on DS of GelMA synthesis. Error bars indicate the relative standard deviations of three independent measurements (n = 3). (**A**) pH transition kinetics during the reaction. (**B**) DS versus MAA/gelatin ratio. DS was obtained from TNBS assay. DS increased much more linearly as the MAA/gelatin ratio increased from 0.0125/1 to 0.1/1 mL/g relative to the conventional method. (**C**) ^1^H NMR verification. Peaks correspond to acrylic protons (2H) of methacrylamide grafts of lysine groups (a) and those of hydroxyl lysine groups (b), methylene protons (2H) of unreacted lysine groups (c), methyl protons (3H) of methacrylamide grafts (d), acrylic protons (2H) of methacrylated grafts of hydroxyl groups (e), and methyl protons (3H) of methacrylated grafts of hydroxyl groups (f).

The dependence of GelMA DS on the MAA concentration was next investigated in order to identify the range for DS controllability and to compare the results with those obtained by other synthesis methods with regards to the MAA supply. The feed ratio was varied from 0.012 to 0.2 ml of MAA per gram of gelatin, which corresponds to 0.265–4.4 molar ratios of the MAA over the amino group. As shown in Fig. 4A, when a larger MAA amount was used, a lower pH was observed during synthesis, as the by-product of methacrylic acid is proportional to the amount of MAA consumed. Figure 4B summarizes the DS results based on different feed ratios compared with literature values^33^,^48^,^50^. Our present findings agree well with our previous identification of a streamline approach (0.1 M CB with pH adjustment)^48^, with the additional advantage that no pH adjustment is required in our improved reaction scheme. As a result, the optimized scheme enables simple, facile one-pot GelMA synthesis under conditions of a 0.25 M CB buffer concentration and initial pH adjustment to 9 with a similar effect on DS as sequential GelMA synthesis under conditions of multistep pH adjustment and MAA addition every 30 min. Moreover, in the present study, the DS results for the MAA/gelatin feed ratio from 0.012 to 0.05 mL/g increased linearly, showing better controllability of DS, compared to conventional method where the relationship is less controllable^33^,^50^. In particular, we observed that methacrylation of lysine groups almost reached saturation around 0.1 mL/g. In Fig. 4C, additional peaks were observed around 5.6 ppm and 6.1 ppm (peak e) in the ^1^H NMR spectrum of the 0.2 mL/g (MAA/gelatin) sample. These peaks can be attributed to partial methacrylation of the hydroxyl groups of gelatin, which occurs when a high molar excess of MAA is supplied^18^,^45^,^48^.

**Figure 5:**
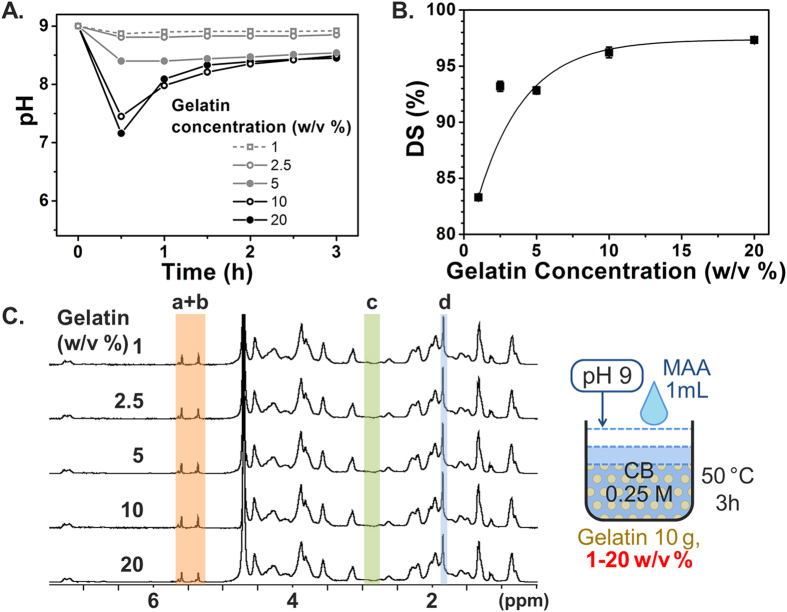
Effect of gelatin concentrations on GelMA synthesis. Error bars indicate the relative standard deviations of three independent measurements (n = 3). (**A**) pH transition kinetics during the reaction. (**B**) DS versus gelatin concentration. DS was obtained from TNBS assay. Gelatin solutions at 10 w/v% and above led to a high DS. (**C**) ^1^H NMR verification. Peaks correspond to acrylic protons (2H) of methacrylamide grafts of lysine groups (a) and those of hydroxyl lysine groups (b), methylene protons (2H) of unreacted lysine groups (c), and methyl protons (3H) of methacrylamide grafts (d).

Based on the aforementioned conditions, we further optimized the gelatin concentration, a parameter which was not discussed in the original paper^34^ although 10 w/v% is conventionally used in most GelMA studies. We investigated gelatin concentrations ranging from 1, 2.5, 5, 10, and 20 w/v%, with all samples containing 10 g gelatin. During synthesis, the 1 w/v% gelatin group exhibited highly separated phases of aqueous (gelatin in CB buffer) and organic compounds (MAA). MAA did not disperse evenly, and it appeared to form large oil droplets in the reaction solution. This apparent phase separation could be the result of the low gelatin concentration, and gelatin is known to be a good emulsifier due to its amphiphilic structure^51^. Indeed, it is reported that surface tension decreases with an increase in gelatin concentration^52^, and this surfactant behavior could help MAA to become evenly dispersed in the reaction solution. Based on these characteristic properties of MAA in aqueous suspensions, we observed that a lower gelatin concentration with a higher buffer capacity maintained a more constant pH (Fig. 5A), but the aforementioned strong phase separation resulted in a lower DS compared with the other groups with a higher gelatin concentration (Fig. 5B). Hence, higher gelatin concentrations are favorable due to improved dispersibility taking into account its surfactant behavior. This result was supported by ^1^H NMR analysis, in which small peaks of methylene of the unreacted lysine amino groups still appeared in the 1 w/v% gelatin group, indicating that some of the lysine amino groups did not react with MAA. One possibility is that MAA may be quickly hydrolyzed at the interface between the MAA droplets and water. The DS was almost saturated above 10 w/v% and the highest DS was obtained with the 20 w/v% concentration. In conclusion, a high concentration of gelatin improved the reaction efficacy with MAA due to the improved miscibility of MAA with gelatin. Importantly, this result indicates that MAA’s solubility in the gelatin solution is an important parameter in the MAA-gelatin reaction.

**Figure 6:**
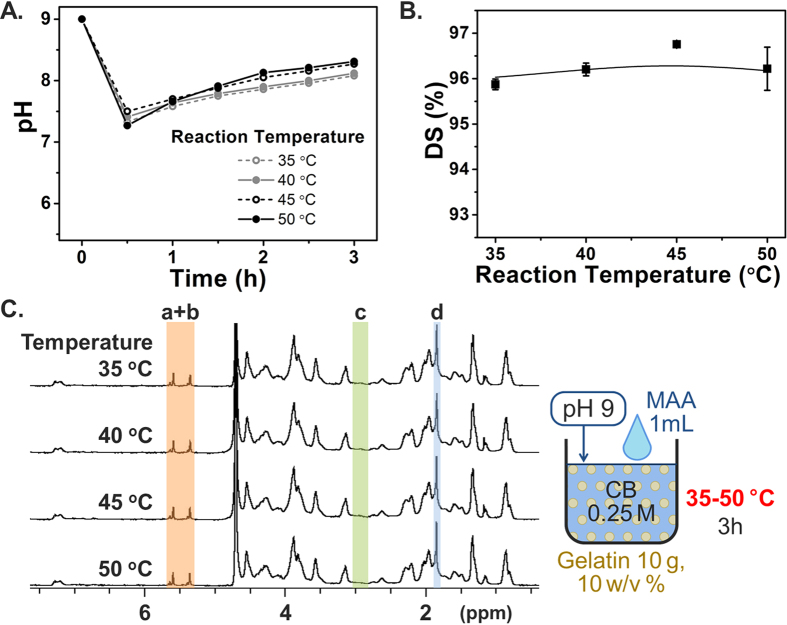
Effect of reaction temperature on GelMA synthesis. Error bars indicate the relative standard deviations of three independent measurements (n = 3). (**A**) pH transition kinetics during the reaction. (**B**) DS versus reaction temperature. DS was obtained from TNBS assay. All conditions led to a high DS. (**C**) ^1^H NMR verification. Peaks correspond to acrylic protons (2H) of methacrylamide grafts of lysine groups (a) and those of hydroxyl lysine groups (b), methylene protons (2H) of unreacted lysine groups (c), and methyl protons (3H) of methacrylamide grafts (d).

Similarly, the effect of reaction temperature on DS was also investigated. In most reports, a single temperature has been used, with the original protocol utilizing a reaction temperature of 50 °C, whereas some studies conducted the GelMA reaction at temperatures between 40 and 60 °C. In our experiments herein, we systematically evaluated the reaction efficacy in lower temperature range to seek possibility in reducing heat supply. Temperatures below 30 °C were excluded because 30 °C is the approximate gelling point of 10% type A gelatin of 250 bloom^53^, and stirring could become inefficient below this temperature. Note that the actual gelling temperature in our case may be slightly lower than that reported in the literature because gelatin of a lower bloom (175 bloom) was used in our experiments. Besides taking into consideration the gelling point, the temperature could also be expected to influence the reaction kinetics, although the effects on the corresponding DS ratio remained to be investigated. Across the evaluated temperatures, the pH transition was similar in the different experimental groups (Fig. 6A), while higher temperatures yielded modestly faster reaction kinetics with more moderate drops in pH during the initial reaction stage. Nevertheless, the DS results in Fig. 6B showed no significant differences across the test groups, with even the 35 °C sample resulting in 96% DS. Collectively, these results support that the GelMA reaction can be conducted at 35 °C with equivalent results to the conventional 50 °C.

**Figure 7:**
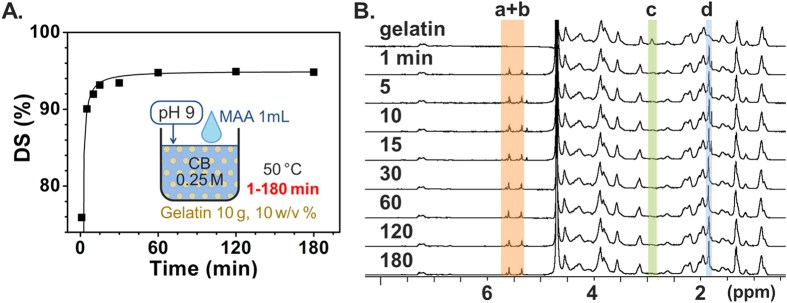
Time-dependent DS monitoring of GelMA synthesis. (**A**) DS versus reaction time obtained from TNBS assay. DS rapidly increased within 1–5 min, with saturation after 1 h. (**B**) ^1^H NMR verification. Peaks correspond to acrylic protons (2H) of methacrylamide grafts of lysine groups (a) and those of hydroxyl lysine groups (b), methylene protons (2H) of unreacted lysine groups (c), and methyl protons (3H) of methacrylamide grafts (d).

The final experimental series was focused on identifying the dependence of GelMA synthesis on the reaction time. Van Den Bulcke *et al*. used a reaction time of 1 h, and subsequent studies used 1–3 h. To investigate the DS as a function of reaction time, sampling was carried out during the standard reaction (0.25 M CB, 0.1 mL/g of MAA/gelatin, 10 w/v% gelatin at 50 °C reaction temperature with initial pH adjustment at 9) at time points 0, 1, 5, 10, 15, 30, 60, 120, and 180 min after MAA addition. The collected samples were immediately quenched, dialyzed, and lyophilized. Figure 7A shows the DS versus reaction time results. It can be seen that the DS continued to increase until 30 min, but after 1 h there was no significant difference among the reaction time points. The ^1^H NMR result in Fig. 7A corroborates the TNBS result, showing a decrease and chemical shift in the methylene peaks of unreacted lysine amino groups (peak c) until and after 30 min, respectively. The immediately lyophilized samples without dialysis showed the exact same tendency in ^1^H NMR peak signatures as shown in Supplementary Figures S2 and S3. Additional peaks also appeared at 1.8, 5.3 and 5.6 ppm, and are attributed to methacrylic acid (the reaction byproduct)^54^. Peaks related to methacrylic acid kept increasing over time, implying progress of MAA hydrolysis, while those corresponding to GelMA product reached a plateau. These results confirm that the reaction between gelatin and MAA is completed within 1 h.

**Figure 8:**
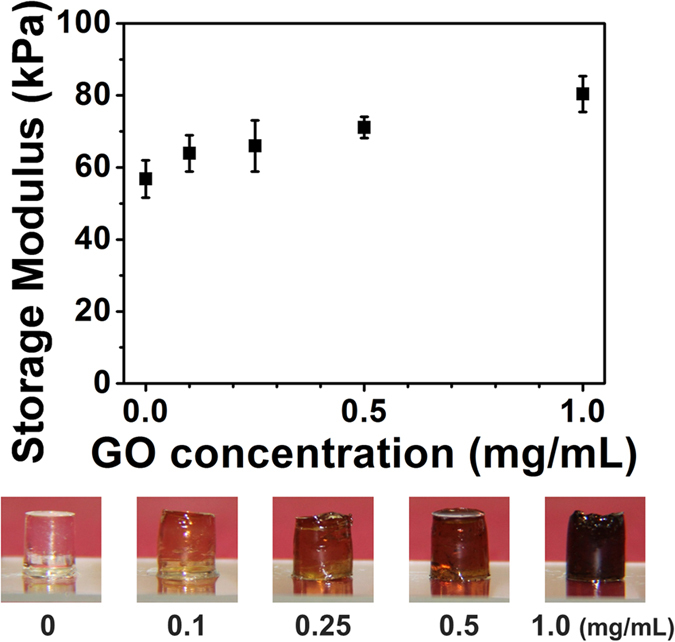
(Top) Storage moduli of GelMA-GO composites with different GO concentrations. (Bottom) Pictures of corresponding bulk hydrogels. The storage modulus of the GelMA-GO composite increased as the concentration of GO increased.

To investigate the mechanical property of the GelMA hydrogel, rheological measurements were conducted. The GelMA samples of different DS (25, 36, 68, 96 and 98%) were dissolved at 30 w/v% in distilled water with 1 w/v% photoinitiator, and irradiated with UV light for 2 min. As shown in Supplementary Figure S4A, the storage modulus showed dependency on DS; ranging from 0.38 ± 0.06 kPa (25% DS) to 86.03 ± 0.96 kPa (98% DS). This trend is in agreement with previous reports^34^,^48^,^50^. To demonstrate GelMA hydrogels with different stiffness values, cylindrical hydrogels were fabricated and a 1.0 N normal force was applied (Supplementary Figure S4B). The hydrogel of the lowest DS (25%) deformed significantly due to low crosslinking density, while hydrogels of higher DS values showed less deformation. These results support the controllability of GelMA hydrogel stiffness. In order to further explore the possibility of GelMA applications, composites of GelMA (96% DS) and graphene oxide (GO) were fabricated at different GO concentrations, specifically 0 (control), 0.1, 0.25, 0.5, and 1.0 mg/mL (Fig. 8). Overall, there was a positive correlation between stiffness and GO concentration, which agrees well with previous reports^29^ and further supports that even highly substituted GelMA can be reinforced with nanomaterials in composite configurations.

In summary, we have comprehensively investigated a facile GelMA synthesis method with different CB buffer molarities, MAA concentrations, gelatin concentrations, reaction temperatures, initial pH adjustment steps, and reaction time, and identified an optimal, one-pot scheme without the need for sequential processing (Fig. 1C, Supplementary Table S1). The results suggest that a simplified synthesis process with a feed ratio of MAA/gelatin at 0.1 mL/g in 0.25 M CB buffer (pH 9) produces GelMA with nearly complete substitution within 1 h. Most previous studies on GelMA synthesis set the reaction temperature at 50 °C with gelatin concentration at 10 w/v%. Additionally, the results presented herein show the possibility of obtaining GelMA with a high DS at reaction temperature of 35–50 °C or higher gelatin concentration of 10–20 w/v%. The synthesized GelMA exhibited gelling properties by photo-crosslinking and its stiffness was controlled by its DS and the amount of a composite additive. Our one-pot GelMA synthesis method yields a GelMA with a controllable DS and is simplified in a controllable manner and is less laborious and more efficient compared to the conventional methods.

## Methods

### GelMA synthesis

The detailed experimental procedure has been described previously^48^. In short, type A gelatin (175 bloom) derived from porcine skin tissue was dissolved in CB buffer (0.1 M buffer comprising 3.18 g sodium carbonate and 5.86 g sodium bicarbonate in 1 L distilled water), and the pH was adjusted with 5 M sodium hydroxide or 6 M hydrochloric acid. Subsequently, MAA (94%) was added to the gelatin solution under magnetic stirring at 500 rpm. The reaction proceeded for 3 h, and then the pH was readjusted to 7.4 to stop the reaction. After being filtered, dialyzed, and lyophilized, the samples were stored at −20 °C until further use. The standard conditions of the synthesis were: CB buffer at 0.25 M, initial pH adjustment at pH 9, MAA amount at 0.1 mL per gram of gelatin concentration at 10 w/v%, reaction temperature at 50 °C and reaction time for 3 h.

In performing detailed characterization of the synthesized GelMA scheme, the following experimental parameters were investigated: CB molarities (0.1, 0.25, 0.5, 0.75, and 1 M), initial pHs (pH 8, 9, 10, and 11), MAA/gelatin feed ratios (MAA/gelatin: 0.0125, 0.25, 0.5, 0.1, and 0.2 mL/g), gelatin concentrations (1, 2.5, 5, 10, and 20 w/v%) and reaction temperatures (35, 40, 45, and 50 °C).

### Reaction Kinetic Experiments

To investigate reaction efficacy, reaction kinetic experiments were performed by sampling the reaction solution at different reaction time points, namely, 0, 1, 5, 10, 15, 30, 60, 120, and 180 min after MAA addition. Each sample of 10 mL was immediately quenched with a 5- to 10-fold amount of water, dialyzed and lyophilized for the determination of DS. For supplemental inspection, 300 μL of the reaction solution was taken at each time point, immediately frozen (without dialysis) at -80 °C, and lyophilized for ^1^H NMR measurement.

### Determination of GelMA DS

To quantify the DS, TNBS assay was performed, as previously described^48^. Briefly, GelMA and gelatin samples were separately dissolved at 1.6 mg/mL in 0.1 M sodium bicarbonate buffer. Then, each sample solution was mixed with 0.01% TNBS solution (in 0.1 M sodium bicarbonate buffer) both at 0.5 mL and then was incubated for 2 h. Next, 0.25 mL of 1 M hydrochloric acid and 0.5 mL of 10 w/v% sodium dodecyl sulfate were added to stop the reaction. The absorbance of each sample was measured at 335 nm. The glycine standard curve was then plotted to determine the amino group concentration, with sample solutions prepared at 0, 8, 16, and 32 μg/mL.

To verify the substitution, ^1^H NMR measurement was also carried out, as previously described^48^. GelMA samples were separately dissolved at around 50 mg/mL in deuterium oxide, and the chemical shift of each sample was measured.

### GelMA-GO composite preparation

GelMA samples of 96% DS were mixed with distilled water (30w/v%), containing GO at 0 (control), 0.1, 0.25, 0.5 or 1.0 mg/mL. The mixtures were subsequently applied ultrasonication (S 60H, 150 W; Elma Schmidbauer) for 1 h to obtain a suspension with good dispersity.

### Storage Modulus Measurement of GelMA

GelMA samples (30 w/v% in distilled water) were prepared at different DS (25, 36, 68, 96 and 98%). The GelMA solutions or aforementioned GelMA-GO composite suspensions were then added with 2-hydroxy-4’-(2-hydroxyethoxy)-2-methylpropiophenone (I2959) at 1 w/v%.

To analyze the mechanical properties of the hydrogels, frequency-sweep measurements in MCR 501 (Anton Paar) were performed similarly as in the previous method^48^. Briefly, 140 μL of each sample was cured (365 nm, 150 mW/cm^2^) between the measurement device and transparent glass plate, at 37 °C. UV exposure times were 2 min for GelMA solutions, and 8 min for GelMA-GO composite suspensions to ensure the complete crosslinking through the turbid hydrogel. A measurement device of 25 mm cone-plate geometry with a cone angle of 2 degrees was used for frequency-sweep measurement at 2% strain amplitude at an oscillation frequency of 0.1–10 Hz within the linear viscoelastic region.

### Demonstration of GelMA Hydrogel Deformation

Cylindrical samples were fabricated with GelMA solutions (30w/v% with 1 w/v% I2959). A volume of 200 μL of each GelMA solution in silicone tube molds (inner diameter at 6.0 mm) was photo-crosslinked by UV irradiation for 6 min. In order to demonstrate deformation of the GelMA hydrogels, a 10 mm parallel-plate was utilized to apply a normal force (1 N) to each GelMA hydrogel. All the chemicals were purchased from Sigma-Aldrich.

## Additional Information

**How to cite this article**: Shirahama, H. *et al*. Precise Tuning of Facile One-Pot Gelatin Methacryloyl (GelMA) Synthesis. *Sci. Rep.* **6**, 31036; doi: 10.1038/srep31036 (2016).

## Supplementary Material

Supplementary Information
