# Supplementary material for: Precise Tuning of Facile One-Pot Gelatin Methacryloyl (GelMA) Synthesis
Source: Sci Rep. 2016 Aug 9;6:31036. doi: 10.1038/srep31036 (PMC4977492; doi:10.1038/srep31036)
Supplement: Supplementary Information [file srep31036-s1.pdf]

Electronic Supplementary Information for

Precise Tuning of Facile One-Pot Gelatin Methacryloyl (GelMA) Synthesis

Hitomi Shirahama[1,†], Bae Hoon Lee[1,†], Lay Poh Tan[1,*], Nam-Joon Cho[1,2,*]

[1] School of Materials Science and Engineering, Nanyang Technological University, 50 Nanyang Avenue, Singapore 639798

[2] School of Chemical and Biomedical Engineering, Nanyang Technological University 62 Nanyang Drive, Singapore 637459

[†] These authors contributed equally to this work.

Table S1. Comparison of three different GelMA synthesis methods

Parameters	Buffer and molarity	pH adjustment	MAA/ Gelatin (mL/g)	Gelatin conc. (w/v %)	Reaction time (h)	Reaction temperature (°C)	DS (%)
Conventional Methods	PBS	NA	(0.8-2)/1	10	1-3	50	>85
Sequential Method	CB 0.1 M	pH 9 (multiple)	total 0.1/1 (multiple)	10	3	50	97
Facile One-pot Method	CB 0.25 M	pH 9 (one-time)	0.1/1 (one-time)	10-20	1	35-50	96

Review of previous studies

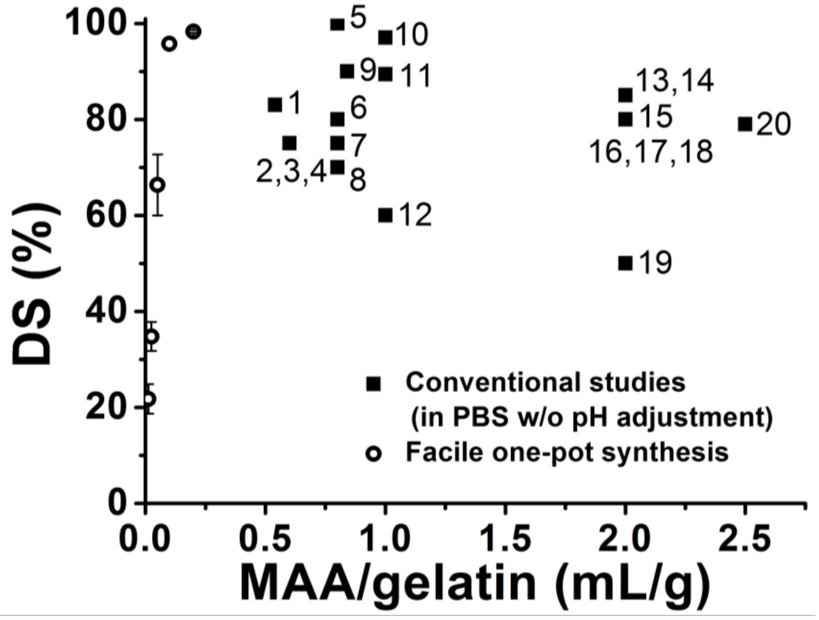

Figure S1. Comparison of DS *versus* MAA/gelatin feed ratio between conventional studies using PBS as a buffer without employment of pH adjustment (closed square) and facile one-pot synthesis (open circle). It indicates that the conventional method of GelMA synthesis has little controllability over DS. Each label corresponds to its related reference.[1-20] Some of the values were obtained from NMR measurement.[5,10,11,13,14,18-20] It is reported that NMR measurement tends to result in a higher DS value compared to colorimetric assays (e.g. TNBS).[21]

NMR results of time-dependent DS monitoring of GelMA synthesis through direct freeze-drying

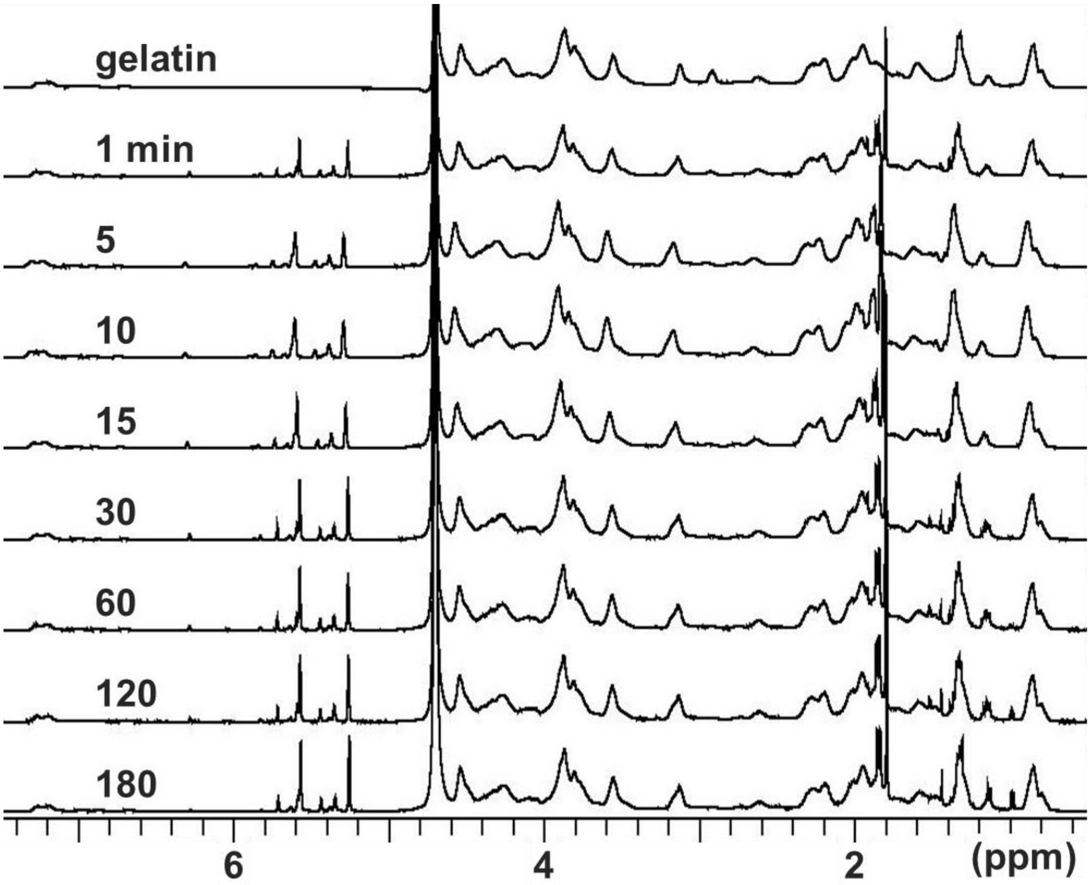

Figure S2. NMR spectra of samples, taken during GelMA synthesis conducted at standard conditions; CB (0.25 M), initial pH adjustment at pH 9, MAA/gelatin (0.1 mL/g), gelatin concentration at 10 w/v %, reaction temperature at 50 °C. Peaks at around 2.8 ppm, which correspond to methylene protons (2H) of unreacted lysine groups, disappeared almost completely after 60 min.

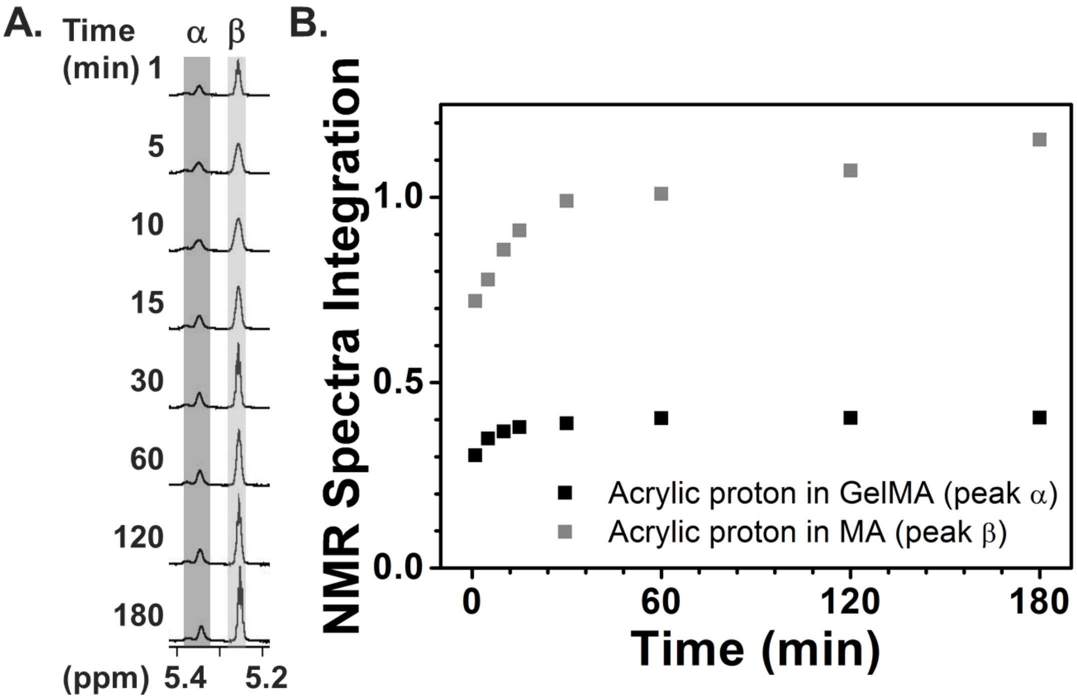

Figure S3. (A) Enlarged spectra of time-dependent NMR samples (Figure S2). Peak α at 5.35 ppm corresponds to an acrylic proton in methacrylated grafts of GelMA, and peak β at 5.25 ppm corresponds to an acrylic proton in the byproduct, methacrylic acid (MA). (B) Integrated NMR peaks of α and β. All the integrations are normalized to peaks of aromatic groups at around 7.2 ppm. Methacrylation reached a plateau after 60 min whereas production of methacrylic acid increased slightly. These results support that the GelMA reaction can be complete within 1 h. Also it suggests that there is a small amount of unreacted MAA after 1 h, being hydrolyzed further.

Mechanical properties of GelMA hydrogels

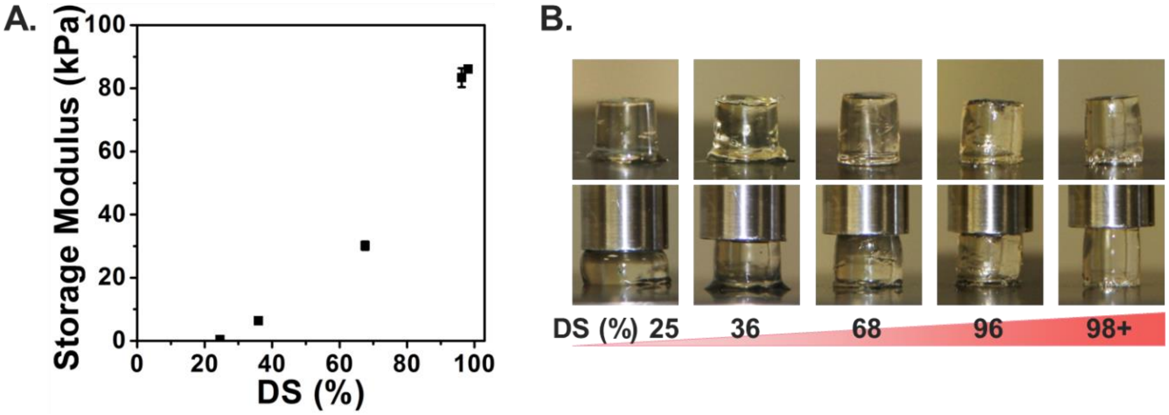

Figure S4. (A) Storage modulus of GelMA samples (30 w/v % in distilled watercontaining 1 w/v % I2959) with different DS. Strain was applied at 2% at frequency of 0.1-10 Hz during the measurement. (B) Demonstration of deformation of cylindrical-shaped GelMA hydrogels, (top) without load and (bottom) with a 1 N normal force applied. Plus (+) in DS denotes additional methacryloylation of the pendant hydroxyl group.

References

1 Daniele, M. A. *et al.* Rapid and Continuous Hydrodynamically Controlled Fabrication of Biohybrid Microfibers. *Adv Funct Mater* **23**, 698-704, doi:10.1002/adfm.201202258 (2013).

2 Schuurman, W. *et al.* Gelatin-Methacrylamide Hydrogels as Potential Biomaterials for Fabrication of Tissue-Engineered Cartilage Constructs. *Macromol Biosci* **13**, 551-561, doi:10.1002/mabi.201200471 (2013).

3 Kaemmerer, E. *et al.* Gelatine methacrylamide-based hydrogels: an alternative three-dimensional cancer cell culture system. *Acta Biomater* **10**, 2551-2562, doi:10.1016/j.actbio.2014.02.035 (2014).

4 Visser, J. *et al.* Endochondral bone formation in gelatin methacrylamide hydrogel with embedded cartilage-derived matrix particles. *Biomaterials* **37**, 174-182, doi:10.1016/j.biomaterials.2014.10.020 (2015).

5 Suo, H., Xu, K. & Zheng, X. Using glucosamine to improve the properties of photocrosslinked gelatin scaffolds. *J Biomater Appl* **29**, 977-987, doi:10.1177/0885328214551009 (2015).

6 Ahadian, S. *et al.* Interdigitated array of Pt electrodes for electrical stimulation and engineering of aligned muscle tissue. *Lab Chip* **12**, 3491-3503, doi:10.1039/c2lc40479f (2012).

7 Zhao, X. *et al.* Photocrosslinkable Gelatin Hydrogel for Epidermal Tissue Engineering. *Adv Healthc Mater*, doi:10.1002/adhm.201500005 (2015).

8 Nikkhah, M. *et al.* Directed endothelial cell morphogenesis in micropatterned gelatin methacrylate hydrogels. *Biomaterials* **33**, 9009-9018, doi:10.1016/j.biomaterials.2012.08.068 (2012).

9 Coimbra, P., Gil, M. H. & Figueiredo, M. Tailoring the properties of gelatin films for drug delivery applications: influence of the chemical cross-linking method. *Int J Biol Macromol* **70**, 10-19, doi:10.1016/j.ijbiomac.2014.06.021 (2014).

10 Jeon, O., Wolfson, D. W. & Alsberg, E. In-situ formation of growth-factor-loaded coacervate microparticle-embedded hydrogels for directing encapsulated stem cell fate. *Advanced Materials* **27**, 2216-2223, doi:10.1002/adma.201405337 (2015).

11 Zhou, L. *et al.* Biomimetic mineralization of anionic gelatin hydrogels: effect of degree of methacrylation. *RSC Advances* **4**, 21997, doi:10.1039/c4ra02271h (2014).

12 Benton, J. A., DeForest, C. A., Vivekanandan, V. & Anseth, K. S. Photocrosslinking of Gelatin Macromers to Synthesize Porous Hydrogels That Promote Valvular Interstitial Cell Function. *Tissue Engineering Part A* **15**, 3221-3230, doi:10.1089/ten.tea.2008.0545 (2009).

13 Pedron, S., Becka, E. & Harley, B. A. Regulation of glioma cell phenotype in 3D matrices by hyaluronic acid. *Biomaterials* **34**, 7408-7417, doi:10.1016/j.biomaterials.2013.06.024 (2013).

14 Pedron, S. & Harley, B. A. Impact of the biophysical features of a 3D gelatin microenvironment on glioblastoma malignancy. *J Biomed Mater Res A* **101**, 3404-3415, doi:10.1002/jbm.a.34637 (2013).

15 Nichol, J. W. *et al.* Cell-laden microengineered gelatin methacrylate hydrogels. *Biomaterials* **31**, 5536-5544, doi:10.1016/j.biomaterials.2010.03.064 (2010).

16 Bae, H. *et al.* Cell-laden microengineered pullulan methacrylate hydrogels promote cell proliferation and 3D cluster formation. *Soft Matter* **7**, 1903-1911, doi:10.1039/C0SM00697A (2011).

17 Aubin, H. *et al.* Directed 3D cell alignment and elongation in microengineered hydrogels. *Biomaterials* **31**, 6941-6951, doi:10.1016/j.biomaterials.2010.05.056 (2010).

18 Fathi, A. *et al.* Enhancing the mechanical properties and physical stability of biomimetic polymer hydrogels for micro-patterning and tissue engineering applications. *European Polymer Journal* **59**, 161-170, doi:10.1016/j.eurpolymj.2014.07.011 (2014).
19 Wang, T., Lai, J. H., Han, L. H., Tong, X. & Yang, F. Chondrogenic differentiation of adipose-derived stromal cells in combinatorial hydrogels containing cartilage matrix proteins with decoupled mechanical stiffness. *Tissue Eng Part A* **20**, 2131-2139, doi:10.1089/ten.tea.2013.0531 (2014).
20 Koshy, S. T., Ferrante, T. C., Lewin, S. A. & Mooney, D. J. Injectable, porous, and cell-responsive gelatin cryogels. *Biomaterials* **35**, 2477-2487, doi:10.1016/j.biomaterials.2013.11.044 (2014).
21 Hoch, E., Schuh, C., Hirth, T., Tovar, G. E. M. & Borchers, K. Stiff gelatin hydrogels can be photo-chemically synthesized from low viscous gelatin solutions using molecularly functionalized gelatin with a high degree of methacrylation. *J Mater Sci Mater Med* **23**, 2607-2617, doi:10.1007/s10856-012-4731-2 (2012).
